# Netrin-1: A Serum Marker Predicting Cognitive Impairment after Spinal Cord Injury

**DOI:** 10.1155/2022/1033197

**Published:** 2022-04-21

**Authors:** Yan Meng, Shifei Sun, Shengnan Cao, Bin Shi

**Affiliations:** ^1^Neck-Shoulder and Lumbocrural Pain Hospital of Shandong First Medical University, Jinan, Shandong Province, China; ^2^School of Acupuncture-Tuina, Shandong University of Traditional Chinese, Jinan, Shandong Province, China

## Abstract

**Objective:**

Although cognitive impairment has received more attention in recent years as a result of spinal cord injury (SCI), the pathogenic process that causes it is still unknown. The neuroprotective effects of Netrin as a family of laminin-related secreted proteins were discovered. The purpose of this study was to determine the changes of serum Netrin-1 after SCI and its relationship with cognitive impairment.

**Methods:**

96 SCI patients and 60 controls were included in our study. We collected baseline data from all participants, measured their serum Netrin-1 levels, and followed up their cognitive levels 3 months later.

**Results:**

The clinical baseline values between the control and SCI groups were not significantly different (*p* > 0.05). However, the serum Netrin-1 level in the SCI group was significantly lower than that in the control group (528.4 ± 88.3 pg/ml vs. 673.5 ± 97.2 pg/ml, *p* < 0.05). According to the quartile level of serum Netrin-1 level in the SCI group, we found that with the increase of serum Netrin-1 level, the MoCA score also increased significantly (*p* < 0.001), indicating that the serum Netrin-1 level was positively correlated with the MoCA score after SCI. After controlling for baseline data, multiple regression analysis revealed that Netrin-1 remained an independent risk factor for cognitive impairment after SCI (=0.274, *p* = 0.036).

**Conclusions:**

Netrin-1 may be a neuroprotective factor for cognitive impairment, which may serve as a serum marker to predict cognitive impairment after SCI.

## 1. Introduction

Cognitive impairment is a widespread complication following spinal cord injury (SCI), affecting approximately 10-60% of patients [1, 2]. Before the 1940s, only a small percentage of patients could survive for a few weeks after injury, but advances in technology have greatly improved the survival rate of SCI [3, 4]. According to statistics, up to 90% of people can survive for 1 year after injury [5]. The potential cognitive impairment of survivors becomes an important part of SCI rehabilitation. Cognitive decline following SCI is diverse, affecting areas including memory, language, abstract reasoning, attention, emotion, concentration, and problem solving [6–8]. Therefore, in order to improve the quality of life after SCI injury, it is particularly important and urgent to find biomarkers for predicting cognitive impairment and formulate targeted treatment and rehabilitation strategies.

Professor Tessier-Lavigne discovered Netrin-1, a membrane protein-like protein, in 1994, and it was initially recognized as a potent chemotactic molecule involved in axon guidance and cell migration during embryonic development [9, 10]. Netrin-1 consists of 604 amino acids and is encoded by the NTN1 gene, which is highly evolutionarily conserved [11–13]. Netrin-1 is mostly expressed in neurons and oligodendrocytes in animals, and it has lately been linked to angiogenesis, inflammation, tissue remodeling, and cancer [14]. Netrin-1 acts as both an attractor and a repulsor, and its function depends on its receptors [15, 16]. Although Netrin-1 was discovered nearly 30 years, its function is still poorly understood.

Although data on Netrin-1's significance in neurological illnesses and SCI have begun to appear in recent years, its association with cognitive impairment remains uncertain [17]. The goal of this study was to look at how serum Netrin-1 changed in the acute phase after SCI and how that related to cognitive impairment. The study of Netrin-1 as a potential serum marker of cognitive impairment after SCI will be helpful for the treatment and rehabilitation of cognitive impairment after SCI.

## 2. Methods

### 2.1. Study Population

Spinal cord injury (SCI) patients presenting to Neck-Shoulder and Lumbocrural Pain Hospital were enrolled in this study. Inclusion criteria are as follows: age > 18 years old; patients with spinal fractures confirmed by CT examination; seek medical treatment within 24 hours after onset; and volunteer to participate in this study. Exclusion criteria were previous neurological trauma or surgical history, severe organic diseases such as heart, liver, and kidney, unstable vital signs, and death within 3 months of injury. In addition, 60 healthy controls were recruited to voluntarily join the study. The hospital's ethics committee approved our research. Informed consent was given by all patients or family members.

### 2.2. Baseline Data Collection

All participants received a questionnaire to collect baseline data at enrollment. These data included age, gender, smoking, drinking, and whether there was a history of hypertension, diabetes, coronary heart disease, and hyperlipidemia. The collection of clinical baseline data was double-checked to ensure accuracy.

### 2.3. Serum Netrin-1 Level Determination

All participants received fasting venous blood collection within 24 days of enrollment. After whole blood was collected, coagulation was performed for 20 minutes at room temperature. Blood clots were removed after whole blood was centrifuged at 2000 × *g* for 10 minutes in a refrigerated centrifuge. The resulting supernatant was designated serum. The determination of Netrin-1 adopts enzyme-linked immunosorbent assay (ELISA) method, and the antibody adopts commercial product. The specific ELISA steps refer to the instructions and previous reports [18, 19].

### 2.4. MoCA Scale Evaluation

MoCA is a simple tool for screening for cognitive impairment and is intended for use by health professionals only. This 10-minute, 30-point assessment tool assesses different cognitive domains, including attention, concentration, conceptual thinking, executive function, visual construction skills, memory, language, calculation, and orientation. MoCA has so far been available in 46 languages [20]. In this study, we used the MoCA scale to evaluate cognitive function 3 months after SCI. All scale reviewers were uniformly trained to reduce bias.

### 2.5. Statistical Analysis

Continuous variables were expressed as mean ± standard deviation (SD), and categorical variables were expressed as percentages. A normality test was performed on these data. The *t*-test was used to assess group differences for continuous variables when the normal distribution was fitted. Categorical variables were compared using the chi-square test. A trend test was used to evaluate the association of Netrin-1 with MoCA. Statistical significance was established when *p* < 0.05. Statistical analysis was performed using the SPSS 22.0 program package.

## 3. Results

### 3.1. Clinical Baseline Data

The control and SCI groups each had 60 and 96 participants, respectively. Between the two groups, there were no significant differences in age, gender, smoking and drinking habits, or past medical history (hypertension, diabetes, coronary heart disease, and hyperlipidemia) (*p* > 0.05).  Table 1 contains the detailed statistics.

**Figure 1:**
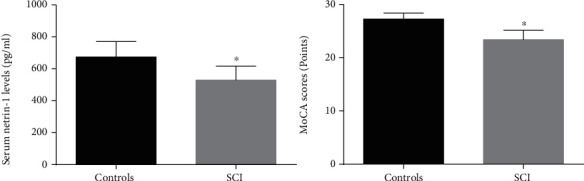
Comparison of serum Netrin-1 levels with MoCA score. ^∗^*p* < 0.05 compared to controls.

However, the MoCA scores of the SCI group and the control group were 23.4 ± 1.8 and 27.3 ± 1.1 points, respectively, and the MoCA score of the SCI group was significantly lower than that of the control group (*p* < 0.001); however, the serum Netrin-1 levels in the SCI group and the control group were 528.4 ± 88.3 pg/ml and 673.5 ± 97.2 pg/ml, respectively, and the serum Netrin-1 level in the SCI (Table 1 and Figure 1) indicates the differences in MoCA scores and serum Netrin-1 levels between the two groups.

### 3.2. Correlation between MoCA and Serum Netrin-1 Level

According to the quartiles of serum Netrin-1 levels in SCI patients, we divided SCI patients into 4 groups, from low to high: Q1, Q2, Q3, and Q4 groups. We also analyzed the MoCA scores of different groups. The *p* for trend test was used to verify the correlation between serum Netrin-1 level trends and MoCA scores. The results showed that serum Netrin-1 level was significantly correlated with MoCA score (*p* < 0.001). The correlation analysis between serum Netrin-1 level and MoCA is shown in Table 2.

### 3.3. Multiple Regression Analysis

We incorporated clinical baseline data into regression equations to analyze potential etiologies affecting cognitive function after SCI. The results (Table 3) showed that age, gender, smoking, drinking, diabetes, hypertension, coronary heart disease, and hyperlipidemia were not the key factors affecting cognitive impairment after SCI. Serum Netrin-1 level could still independently predict cognitive impairment after SCI after adjusting for the above interference factors (*β* = 0.274, *p* = 0.036).

## 4. Discussion

This study was a single-center clinical trial on the hot topic of cognitive impairment following spinal cord injury. The goal of this study was to see if there was a link between serum Netrin-1 and cognitive impairment following a spinal cord injury. The findings revealed that in the acute phase of SCI patients, serum Netrin-1 levels were much lower, and this decline was significantly linked with the MoCA score. The serum Netrin-1 level could predict cognitive damage after SCI independently, according to regression analysis. Our research is the first to show a link between Netrin-1 and cognitive impairment following a spinal cord injury.

For decades, developmental neurobiologists have been searching for chemical molecules with axon guidance mechanisms. In 1994, the first biochemical purification of axonal growth-promoting factors was identified in chicken embryos, named Netrins (Netrin-1 and Netrin-2), a laminin-related molecule involved in axon guidance and cell migration [17, 21, 22]. Netrin-1, the most studied member of the family, is highly conserved during development and is expressed in neuroepithelial cells of the floor plate and ventral region of the spinal cord. In the nervous system, it is mainly expressed in neurons and oligodendrocytes; outside the nervous system, Netrin-1 is expressed in the limb primordia, breast, pancreas, dorsal aorta, and ovary [17, 23, 24]. Netrin-1 has dual functions depending on the receptor. Binding of Netrin-1 alone to DCC results in axonal attraction that recruits intracellular signaling complexes to activate and trigger cytoskeletal rearrangements [25, 26]. However, when un5a D coexists with DCC, Netrin-1 enables the DCC P1 motif to interact with the DB domain of Unc5A D, initiating the Netrin-1 exclusion signaling pathway [25, 27, 28]. Although Netrin-1 has been discovered for many years, its specific regulatory mechanism in vivo has not been fully elucidated.

The role of Netrin-1 in neurological diseases has been widely reported. Evidence suggests that Netrin-1 may be a promising drug candidate for reducing stroke severity and improving prognosis, and its neuroprotective mechanisms may be achieved through regulation of angiogenesis, autophagy, apoptosis, and neuroinflammation [29]. Glioblastoma is one of the most common tumors in neurosurgery, and evidence in recent years suggests that Netrin-1, as an atypical angiogenic ligand, may be involved in the promotion of neovascularization in glioblastoma [30]. Evidence that Netrin-1 is involved in the pathogenesis of Alzheimer's disease has been found in cells, animals, and clinical experiments, indicating that Netrin-1 may be involved in the regulation of neuroplasticity [31–33]. Studies on the regulation of Netrin-1 receptors in Parkinson's disease have also been reported [33–35]. Another study pointed out that serum Netrin-1 concentration decreased after brain injury and could be used as a biomarker of traumatic brain injury [36]. Further animal experiments showed that the mechanism of Netrin-1 protecting traumatic brain injury may be related to its protection of the integrity of the blood-brain barrier [37]. Netrin-1 and its receptor are involved in the maintenance of neural function as an important neural regulatory axis.

The role of Netrin-1 in SCI is beginning to emerge. In order to explore the potential role of Netrin-1 after SCI, Canadian scholars detected the expression of Netrin-1 and receptors in rats after spinal cord amputation. The results showed that Netrin-1 was significantly decreased at the injury site and sustained a low decrease for 7 months and was mainly expressed in neurons and oligodendrocytes immediately adjacent to the lesion [38]. Chinese researchers explored the therapeutic effect of Netrin-1 in a rat model of spinal cord injury in a study. The results show that the motor function of injured rats can be significantly improved, and the mechanism may be achieved by activating the AMPK/mTOR signaling pathway to regulate autophagy [39, 40]. The expression of Netrin-1 protein in the spinal cord of rats with experimental autoimmune encephalomyelitis (EAE) was studied by a Korean research team. Netrin-1 levels in the spinal cord of rats at the height of EAE have been found to be greatly elevated, whereas normal human Netrin-1 is mostly expressed in spinal neurons, oligodendrocytes, astrocytes, and vascular endothelial cells [41]. The regulatory role of Netrin-1 in EAE injury in rats has been discovered, but its research in humans has not been reported yet.

Our study is the first to report the involvement of Netrin-1 in cognitive impairment after SCI. Despite the obvious strengths of our study, the limitations of our study still need to be stated. First, we are not a multicenter large-sample study; second, we have no mechanistic studies involving animal or cell experiments; and finally, our study lacks detection data for Netrin receptors.

## 5. Conclusions

In the acute phase of SCI, serum Netrin-1 levels were much lower, and this was linked to a reduction in cognitive performance. The level of serum Netrin-1 may be a serum marker for predicting cognitive impairment after SCI, and a deeper understanding of its mechanism could modify the prevention and treatment strategy for cognitive impairment after SCI.

## Figures and Tables

**Table 1:** Baseline data from the study population.

	Controls (*n* = 60)	SCI (*n* = 96)	*p* value
Age, years	58.2 ± 6.4	58.6 ± 6.9	0.718
Gender, male/female	49/11	81/15	0.659
Drinking, *n* (%)	41	70	0.539
Smoking, *n* (%)	37	62	0.713
Hypertension, *n* (%)	19	31	0.935
Diabetes, *n* (%)	13	19	0.778
Coronary heart disease, *n* (%)	10	18	0.741
Hyperlipidemia, *n* (%)	26	38	0.643
Netrin-1 (pg/ml)	673.5 ± 97.2	528.4 ± 88.3	<0.001
MoCA (points)	27.3 ± 1.1	23.4 ± 1.8	<0.001

**Table 2:** Relationship between serum Netrin-1 levels and MoCA scores.

Variable	Q1	Q2	Q3	Q4	*p* values
MoCA scores	22.5 ± 1.7	23.1 ± 2.0	23.8 ± 1.9	24.2 ± 1.6	<0.001

**Table 3:** Multiple regression analysis for predicting cognitive impairment in SCI patients.

	Regression coefficient	95% confidence interval	*p* value
Age	0.132	0.107-1.016	0.163
Gender	0.205	0.148-1.098	0.312
Drinking	0.227	0.183-1.034	0.405
Smoking	0.218	0.109-1.105	0.199
Hypertension	0.186	0.124-1.073	0.227
Diabetes	0.260	0.185-1.121	0.174
Coronary heart disease	0.191	0.086-1.062	0.338
Hyperlipidemia	0.243	0.162-1.240	0.081
Netrin-1	0.274	0.113-0.853	0.036

## Data Availability

The study data presented may be made available from the corresponding author upon reasonable request.
